# Promoting Recruitment using Information Management Efficiently (PRIME): statistical analysis plan for a stepped wedge cluster randomised trial within the REstart or STop Antithrombotics Randomised Trial (RESTART)

**DOI:** 10.1186/s13063-017-1840-8

**Published:** 2017-03-01

**Authors:** Richard A. Parker, Christopher J. Weir, Amy E. Maxwell, Rustam Al-Shahi Salman

**Affiliations:** 1 Edinburgh Clinical Trials Unit and Centre for Population Health Sciences, Usher Institute of Population Health Sciences and Informatics, Old Medical School, Teviot Place, Edinburgh, UK; 2 Centre for Clinical Brain Sciences, University of Edinburgh, Edinburgh, UK

**Keywords:** Statistical analysis plan, Complex intervention, Stepped wedge trial, Cluster randomised trial, Recruitment, Study-within-a-trial, Trial-within-a-trial

## Abstract

**Background:**

Promoting Recruitment using Information Management Efficiently (PRIME) is a stepped wedge, cluster randomised trial-within-a-trial of a complex intervention to help sites in the United Kingdom to attain their own target number of participants to recruit to the REstart or STop Antithrombotics Randomised Trial (RESTART, ISRCTN71907627).

**Methods:**

Seventy-two hospital sites had opted into PRIME and were randomly allocated (using a computer-generated block randomisation algorithm, stratified by hospital location) to one of 12 months in which a complex intervention would be delivered. All sites began in the control state. The primary outcome is the total number of patients randomised into RESTART per month per site, which will be analysed in a negative binomial generalised linear mixed model. Secondary outcomes include the proportion of sites using stroke databases to identify potentially eligible patients before PRIME, frequency of using bespoke stroke audit data exports during PRIME, barriers to recruitment in PRIME, barriers to using the bespoke stroke audit data exports, and disadvantages of the bespoke stroke audit exports identified by PRIME sites. PRIME began in September 2015. The last intervention will be delivered in August 2016. Six-month follow-up will be complete in February 2017. This statistical analysis plan was written and submitted for publication before all sites received the PRIME intervention and before outcome data were known.

**Discussion:**

Final results of PRIME will be analysed and disseminated in 2017.

**Trial registration:**

Northern Ireland Hub for Trials Methodology Research SWAT repository (SWAT22).

## Background

The REstart or STop Antithrombotics Randomised Trial (RESTART, ISRCTN71907627) is an on-going randomised controlled trial (RCT) of secondary prevention after stroke due to intracerebral haemorrhage (ICH). Promoting Recruitment using Information Management Efficiently (PRIME) is a stepped wedge, cluster randomised trial of a complex intervention to help RESTART sites increase their recruitment and attain their own target number of participants. Seventy-two hospital sites that were active in RESTART in 2015 opted into PRIME, so the trial can be considered as a study-within-a-trial (SWAT).

### Intervention

The complex intervention involves a recruitment coordinator discussing recruitment strategies with the principal investigator (PI) and RESTART coordinator at each site, providing software for each site to extract from their own stroke audit data lists of patients who were potentially eligible for RESTART, and a second teleconference to review progress 6 months later. The key components of the intervention are:

- Discussion on how staff at the site have been finding RESTART to date
- Review of the recruitment commitment made at the site initiation visit and how many patients they have recruited
- Review of the information in the pre-review questionnaire about recruitment completed prior to the recruitment review
- Explanation of the availability of bespoke stroke audit data exports and examples provided of how the pilot sites effectively used them
- Review of the use of a template invitation letter for approaching prevalent patients and discussion about how they can be used effectively in conjunction with the bespoke stroke audit data exports
- Review of the opportunities to recruit inpatients and outpatients at the hospital site
- Sharing of what other methods top recruiting sites have been using to identify, consent and randomise patients
- After the review, an email is sent to all the collaborators at the site summarising what had been discussed at the review, providing attendance certificates, and giving instructions for running the relevant bespoke stroke audit data exports

### Study design

A stepped wedge design was chosen because it would have been very difficult to apply the intervention across all intervention clusters simultaneously in a standard, parallel-group, cluster randomised trial design. By allowing a phased introduction of the intervention in a stepped wedge design, this meant that it was only necessary to have a single PRIME coordinator to implement a tailored intervention at each individual site; and so it was logistically and practically easier to implement the intervention. Furthermore, all sites will have been given a potentially efficacious intervention by the end of the trial, whilst the expected burden on these sites was perceived to be low. There was also no foreseeable negative impact on patients in terms of harm or burden due to the intervention. In addition, we would expect that the intracluster correlation coefficient might be relatively high in this trial as a result of high variability in average recruitment rates between sites; leading to possibly greater statistical power for the stepped wedge design compared to a standard cluster trial design [1].

PRIME has a closed-cohort, stepped wedge design whereby all participating sites are included from the start through to the end of the trial, and we are interested in assessing the recruitment rate within fixed time periods [2]. The timing of the recruitment review was randomised, stratified by hospital location (Scotland versus England/Wales). This involved using computer-generated block randomisation to randomly allocate the 72 sites to groups of six with 12 different months for the recruitment review in one calendar year, stratified by hospital location. The investigators, recruitment coordinator, and sites were all blinded to the timing of the recruitment review until this had to be revealed in order to organise the recruitment review. Members of staff at each site were asked to complete questionnaires before the recruitment review and 6 months afterwards enabling us to characterise sites, identify barriers to recruitment, and determine any perceived disadvantages to applying the intervention. Further details about the PRIME trial design, including the sample size calculation, can be found in the trial protocol paper [3]. In this article we describe the statistical analysis plan which outlines how data analysis will be conducted at the end of the trial. The statistical analysis plan was finalised and approved by the co-authors on 4 April 2016.

### Outcomes

#### Primary outcome

The primary outcome is the total number of patients randomised into the RESTART trial per month per site.

#### Secondary outcomes

We will collect secondary outcomes, from the information supplied in the questionnaires completed before, at, and 6 months after the recruitment review. These include:

1. Number of sites in PRIME that routinely used stroke databases to identify potentially eligible patients before receiving the recruitment review
2. Number of sites in PRIME that used the bespoke stroke audit data exports, and the frequency of their use in the 6 months after the recruitment review
3. Barriers to recruitment in PRIME, identified by sites in the questionnaires completed before, at, and 6 months after the recruitment review
4. Barriers to using the bespoke stroke audit data exports identified by PRIME sites at the 6-month follow-up review
5. Disadvantages of the bespoke stroke audit exports identified by PRIME sites at the 6-month follow-up review

### General analysis principles

Analyses will include all sites as randomised unless otherwise stated below. We will include all 72 sites that agreed to take part in PRIME regardless of the circumstances of implementation of the recruitment review or subsequent withdrawal. Any sites for which the recruitment review was not implemented at the correct time or failed to be implemented will still be included in an intention-to-treat analysis provided that outcome data are available.

There will be no imputation of missing data: missing values will be left as missing for all statistical analyses.

In general, categorical data will be presented using counts and percentages, whilst continuous variables will be presented using the mean, median, standard deviation (SD), minimum, maximum, first quartile, third quartile, and number of sites with a response (*n*).

Outliers will be identified by viewing boxplots or histograms and will be queried at the data checking stage if an error is suspected. All analyses will include outliers as standard.

All statistical tests and confidence intervals will be two-sided. Ninety-five percent confidence intervals will be presented with the significance of *p* values assessed based on a 5% significance level. No adjustment for multiplicity will be made in any of the analyses. There is a single primary outcome. The secondary outcomes are exploratory, and interpretation of their analyses will be suitably cautious.

There will be no interim analysis of the trial data and the final analysis will be performed after all follow-up data have been collected.

## List of analyses

### Recruitment of sites and retention

**Fig. 1 ﻿:**
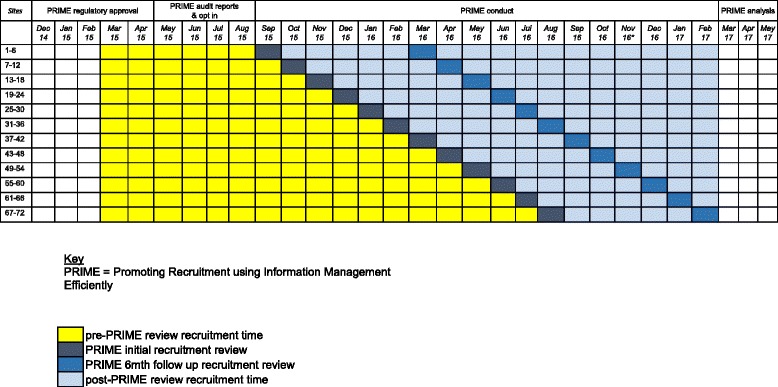
Schematic representation of the PRIME study

An adapted Consolidated Standards of Reporting Trials (CONSORT) diagram will be constructed, as recommended by Davey et al. [4] including details of the number of sites recruited, number of site dropouts (if any, with reasons), number receiving the intervention, number receiving the intervention and providing complete follow-up data, number included in the primary analysis, and any issues with intervention delivery or timing. In particular, we will compare the recruitment and data collection details across the 12 randomised groups of six sites. A schematic representation of the actual study design will be reported, similar to that shown in Fig. 1.

### Descriptive analysis of baseline characteristics

Site characteristics will be summarised stratified by randomised allocation group and overall. No formal statistical testing will be performed at this stage. The site characteristics will include:

1. Site location (Scotland versus England/Wales)
2. Average number of patients randomised per month in the first 6 months of the trial, before any of the sites were scheduled to implement the intervention (March 2015 to August 2015)
3. Proportion of stroke inpatients, who are suitable for follow-up, seen in clinic after hospital discharge
4. Number and percentage of sites that had approached patients looked after by their stroke unit in the past to invite them back to clinic with a view to recruit them to RESTART
5. Number and percentage of sites with complete and accurate stroke audit data
6. Number and percentage of sites already routinely using the stroke audit data to recruit to RESTART
7. Number and percentage of sites using screening logs as a source of information to identify eligible RESTART patients
8. Number and percentage of sites using a database other than the stroke audit as a source of information to identify eligible RESTART patients
9. Number and percentage of sites using another source of information (other than databases or screen logs) to identify eligible RESTART patients
10. Number and percentage of sites already using other methods to boost recruitment
11. Number and percentage of sites already identifying barriers to finding suitable patients to recruit to RESTART

### Primary analysis of the primary outcome

**Fig. 2:**
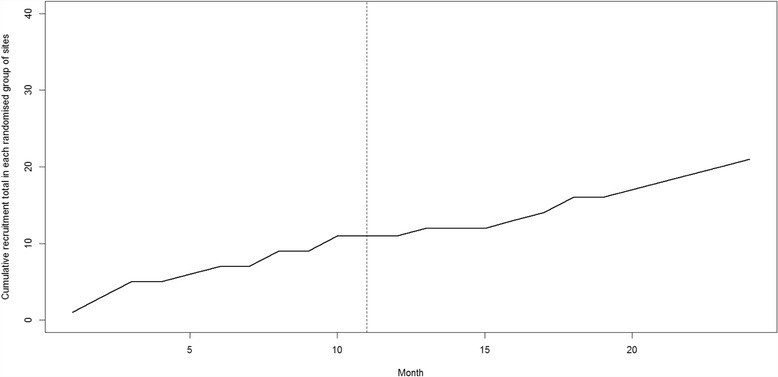
Example of the line plot that will be drawn to show changes in cumulative recruitment rate over time in each randomised group of sites (this plot does not contain any real data)

**Fig. 3:**
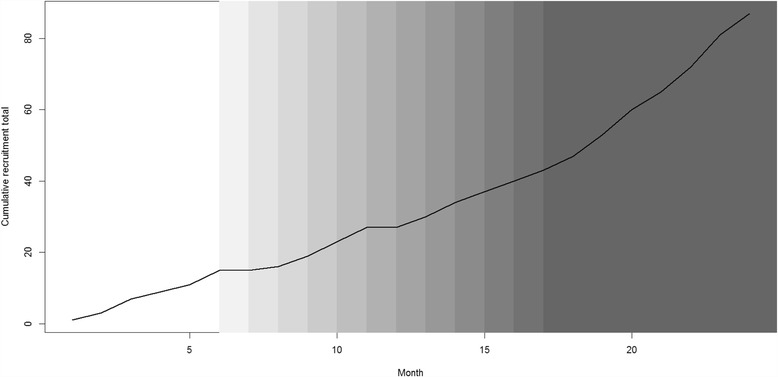
Example of the line plot that will be drawn to show the change in cumulative recruitment rate over time for all sites (this plot does not contain any real data)

To assess how the primary outcome changes over time, the cumulative total randomisation rate since the start of the PRIME study will be plotted in a graph for each randomised group of six sites, with a vertical dashed line to indicate when the sites cross over into the intervention phase. This will be done separately for each of the 12 randomised groups so that we can clearly see how the total recruitment rate for each of the groups changes over time with reference to the start of the intervention (Fig. 2). To provide an overall assessment, we will also plot the cumulative total recruitment rate across all sites with shading to indicate how many sites had received the intervention up to that point (Fig. 3).

Informed by a published method to model recruitment [5], we will fit a negative binomial generalised linear mixed model (GLMM) to the primary outcome, adjusting for the fixed effects of time since start of study (in months), season (December/January (when recruitment rates in RESTART have been low) versus all other months), site location (Scotland versus England/Wales), and an indicator variable for whether the PRIME complex intervention has been implemented or not (according to the planned randomisation schedule). Site will also be included as a random effect in the model. For the primary analysis, the secular change in recruitment rate over time will be adjusted for in the models using a single continuous linear term, unless the graphs of recruitment rate over time strongly indicate otherwise.

The results will be expressed as rate ratios with 95% confidence intervals. The number of decimal places will be determined by the ‘rule of four’ [6]. The estimated time effect will also be reported from the fitted model.

The primary analysis will follow an ‘as-randomised’ principle, which means that the data will be analysed according to the randomised timing of the recruitment review rather than the actual time that the recruitment review occurred. In addition, for the primary analysis, all sites will be included regardless of any site withdrawals and/or compliance with the PRIME trial procedures.

### Secondary analyses of the primary outcome

There will be four separate secondary analyses of the primary outcome, which will each involve using the same negative binomial GLMM methodology as described for the primary outcome with the following modifications:

1. The negative binomial GLMM will additionally include model terms representing the length of time after the randomised introduction of the intervention to assess whether there is a time trend in the effect of the complex intervention on recruitment rate. This will consist of including categorical dummy variables in the model representing the categories ‘3-6 months after recruitment review’ and ‘more than 6 months after review’; with ‘first 3 months after review’ as the reference category
2. A per-protocol ‘actual times’ analysis will be performed: based on the actual times of the recruitment review, and only including those sites that continued to be active in both the RESTART and PRIME trials until the end of the PRIME study period
3. The analysis will be restricted to use only data collected within the rollout period (i.e. the period of time when there were sites in both intervention and control conditions). This is to reduce the risk of confounding due to secular changes over time [2, 4], at the expense of a reduced sample size. An additional site-level explanatory variable will be included in the GLMM: the average number of people randomised per month before the PRIME rollout phase (i.e. before the first site received the intervention)
4. We will assess the sensitivity of the model results with respect to different ways of specifying the adjustment for secular trend in the models. For example, we may model time using a categorical factor variable or by using spline functions that may more appropriately reflect the change in recruitment rates observed in the graphical analysis of primary outcome (i.e. in plots such as those depicted in Figs. 2 and 3)

### Primary outcome analysis assumptions

All analyses of the primary outcome assume that the evaluation will not be confounded by other simultaneous methods to boost recruitment, and that the recruitment coordinator’s effectiveness will remain constant over time (i.e. it will not be diluted by the increasing number of sites that she will support over time, or influenced by seasonal changes in trial recruitment).

### Secondary outcome analyses

The PI, delegated physicians and/or research staff at each RESTART hospital site will complete a 6-month post-recruitment review questionnaire and the responses will be analysed descriptively. For questions that require a ‘yes’ or ‘no’ answer, the results will be summarised as number (percentage). Specifically, we will report:

1. The number and percentage of sites generating and using the stroke audit data exports
2. The number and percentage of sites experiencing any problems running or using the audit reports out of all those answering ‘yes’ to the question of whether they extracted and used the stroke audit data exports

We will also report the number and percentage of sites that had used a template invitation letter to invite potential RESTART patients to clinic before the recruitment review took place, as recorded in the pre-recruitment review questionnaire. (The template invitation letter was originally sent out to RESTART sites in a substantial amendment notification (Ref. 12/SS/0138, amendment REC REF AM18/1) in May 2015.)

For questions in the 6-month post-recruitment review questionnaire generating continuous data, results will be summarised across all sites in the form of mean, median, SD, minimum, maximum, interquartile range (IQR) and number of sites with a response (*n*). In particular, we will report summary statistics for:

1. How far back the site ran the reports to
2. How many times the site ran the reports
3. The number of patients identified by the audit reports
4. The number of eligible patients identified by the audit reports
5. The percentage of patients who were actually eligible out of all those identified by the audit reports
6. The number of eligible patients who the site contacted
7. The percentage of patients who were actually contacted out of all those eligible
8. The number of eligible patients responding
9. The percentage of patients responding out of all those contacted
10. The number of eligible patients who came back to clinic
11. The percentage of patients coming back to clinic out of all those responding
12. The number of eligible patients declining to come back to clinic
13. The percentage of patients declining to come back to clinic out of all those responding
14. The number of patients who were randomised as a result of being identified by the stroke audit data exports
15. The percentage of patients who were randomised out of all patients who came back to clinic

Question 10 of the questionnaire asks ‘Do you think that the reports were useful in identifying potentially eligible patients?’ and answers are on a 5-point Likert scale ranging from ‘Strongly Agree’ to ‘Strongly Disagree’. For this outcome, the number and percentage of sites in each category will be reported; and a bar chart will be used to display the data graphically.

The final three questions require mainly qualitative responses and so, if appropriate, frequency tables will be produced to show the most frequent responses.

The number of complaints from PIs and site co-ordinators about the interventions will be reported, along with the number of sites making at least one complaint. The qualitative reasons for these complaints will also be listed and categorised in a frequency table if appropriate.

We will record any costs of implementing the interventions (for the stroke audits, local RESTART investigators, and participants in RESTART) if available, and we will report corresponding summary statistics (e.g. mean and SD).

## Reporting

We will report PRIME in a manner consistent with the adaptation of the CONSORT reporting guidelines for cluster randomised trials [7], the guidelines for reporting embedded recruitment trials [8], and the recommendations for reporting stepped wedge trials proposed by Hemming et al. [1] and Davey et al. [4].

## Abbreviations

GLMM: Generalised linear mixed model
ICH: Intracerebral haemorrhage
PRIME: Promoting Recruitment using Information Management Efficiently
RESTART: REstart or STop Antithrombotics Randomised Trial
SD: Standard deviation
